# A Survey of Archaeal Restriction–Modification Systems

**DOI:** 10.3390/microorganisms11102424

**Published:** 2023-09-28

**Authors:** Brian P. Anton, Richard J. Roberts

**Affiliations:** New England Biolabs, Ipswich, MA 02127, USA

**Keywords:** archaea, restriction–modification, methyltransferase, SMRT sequencing, REBASE

## Abstract

When compared with bacteria, relatively little is known about the restriction–modification (RM) systems of archaea, particularly those in taxa outside of the haloarchaea. To improve our understanding of archaeal RM systems, we surveyed REBASE, the restriction enzyme database, to catalog what is known about the genes and activities present in the 519 completely sequenced archaeal genomes currently deposited there. For 49 (9.4%) of these genomes, we also have methylome data from Single-Molecule Real-Time (SMRT) sequencing that reveal the target recognition sites of the active m^6^A and m^4^C DNA methyltransferases (MTases). The gene-finding pipeline employed by REBASE is trained primarily on bacterial examples and so will look for similar genes in archaea. Nonetheless, the organizational structure and protein sequence of RM systems from archaea are highly similar to those of bacteria, with both groups acquiring systems from a shared genetic pool through horizontal gene transfer. As in bacteria, we observe numerous examples of “persistent” DNA MTases conserved within archaeal taxa at different levels. We experimentally validated two homologous members of one of the largest “persistent” MTase groups, revealing that methylation of C(m^5^C)WGG sites may play a key epigenetic role in Crenarchaea. Throughout the archaea, genes encoding m^6^A, m^4^C, and m^5^C DNA MTases, respectively, occur in approximately the ratio 4:2:1.

## 1. Introduction

Restriction–modification (RM) systems are one of the best-known defense systems used by prokaryotes to prevent phage infection [1,2]. They comprise a restriction enzyme (REase) that cleaves unmodified DNA and a DNA methyltransferase (MTase) that modifies DNA to block cleavage by the cognate REase. There are four main types of such systems. Type I systems employ three subunits acting in complex, where the R subunit is responsible for restriction, the M subunit is responsible for methylation, and the S subunit is responsible for recognizing the specific DNA sequence that is to be modified or cleaved. Type II systems usually contain two independent enzymes, an REase and an MTase, both of which must recognize and target the same DNA sequence. However, in some Type II systems (designated Type IIG), the MTase and REase activities are encoded in the same polypeptide. Type III systems, like Type I systems, consist of subunits that must act as complexes: an MTase (Mod) subunit that is also solely responsible for sequence recognition and an REase (Res) that must complex with the Mod subunit to cleave unmodified sequences. Type IV systems, also found in many prokaryotes, comprise only an REase that cleaves methylated DNA. Examples of all four types of systems are found in both bacteria and archaea.

REBASE is a comprehensive database of sequence and experimental information about RM systems, drawing information from all fully sequenced microbial genomes deposited in GenBank [3,4]. Methylome data derived from Single-Molecule Real-Time (SMRT) sequencing are also included, enabling the assignment of target sites to MTases and their companion REases. Such assignments are then propagated to homologous enzymes in other organisms for which no experimental data are available. While nanopore sequencing is also capable of detecting DNA methylation, the accuracy of de novo motif calling, particularly for motifs with m^6^A and m^4^C, is currently lower than for SMRT sequencing [5,6]. As a result, relatively little nanopore-based microbial methylation data have been deposited in REBASE to date. We expect this to change as methods continue to improve.

RM systems of bacteria have been far more extensively studied than those of archaea. There have been numerous studies surveying different types of RM systems that largely or exclusively focus on bacteria, many of which use REBASE as source data. Such studies have focused on such topics as Type I systems with recombining S subunits [7], phase-variable Type I systems [8], phase-variable Type III systems [9], solitary REase genes [10], conserved (“persistent”) MTases [11], and the association of RM systems with mobile elements and genome rearrangements [12]. Surveys of RM systems found specifically in archaea are fewer, with the largest being a study in Halobacteria [13]. This review examines more broadly the RM systems of archaea, for which there is relatively little experimental data about restriction and REases. Owing to methylation-sensitive sequencing techniques such as SMRT sequencing, however, our knowledge of DNA methylation and MTases in archaea is improving. There are currently 519 complete DNA sequences for archaeal genomes and SMRT methylation data are available for 49 of these.

## 2. Materials and Methods

### 2.1. Identification of Genomes

We retrieved a list of accession numbers of all genome sequence files stored in the REBASE database [3] and grouped together different accession numbers associated with the same strain (*n* = 59,327 strains). From this list, we first retrieved all genome sequences that had been taxonomically curated by NCBI and were stated to belong to the domain Archaea (*n* = 697 strains). We next retrieved all sequences, regardless of taxonomic assignment, that had not originated with NCBI (*n* = 1417 strains). The latter set was manually curated to identify the archaea (*n* = 15 strains), and these were combined with the NCBI set for a total of 712 strains.

This set was further parsed to remove genomes whose sequence was not complete at the time of accession. Of the 712 strains, 487 were flagged as “complete genome” in the GenBank definition line and retained. From the other 225 strains, we removed those flagged as whole-genome shotgun data, those where the longest sequence was less than 500 kb, and those where the status of the NCBI genome sequence project was anything less than complete. The remaining strains in the latter set (*n* = 32 strains) were combined with the earlier set for a total of 519 archaeal strains with complete genome data in REBASE. Of these, 49 also had associated methylome data from SMRT sequencing.

### 2.2. Identification and Clustering of Genes

Genomes processed for entry into REBASE were analyzed to identify genes associated with RM systems using the SEQWARE v. 4 software pipeline [14]. We obtained all such genes encoded by the 519 archaeal strains identified above (*n* = 4135 protein sequences). These sequences were clustered to 30% sequence identity using Usearch v11 cluster_fast (*n* = 1034 sequence clusters) [15]. 

### 2.3. Construction of HMM Library

To predict the function of the uncharacterized archaeal proteins, we built a library of 62 HMMs spanning many different RM system-related functions and protein types (Supplementary Materials, Table S1). Protein sequences from which these HMMs were constructed were obtained from REBASE, focusing on experimentally characterized examples, where available, and their close homologs. Of these protein sequences, 462 were DNA MTases (including Type IIG RM proteins) and 202 were of all other functions (REases, S proteins, etc.). Sequences comprising two fused MTase domains were separated into component domains, but other multidomain proteins (Type IIG RM proteins, for example) were left intact. These two groups were separately clustered and visualized in two dimensions using CLANS [16] run under the MPI Bioinformatics Toolkit [17]. The resulting clusters were used to verify and refine the protein sets used for each HMM. Most of the final sets formed visually well-defined clusters in the CLANS analysis.

The protein sets were presumed to comprise functionally similar and/or evolutionarily related groups of proteins. The MTase sets were generally homogeneous in terms of methylation type (m^6^A, m^4^C, or m^5^C) based on experimentally characterized examples. However, protein sequences of MTases conferring m^6^A and those conferring m^4^C can be very similar [18], and four HMMs (b1a, lmoa118-like, nru-like, and b3) were built from sequence sets that included characterized MTases of both types (Supplementary Materials, Table S1). For the purpose of classifying based on methylation type (used in the tables in this work), the HMMs b1a, lmoa118-like, and nru-like were all considered to be m^6^A, and b3 was considered to be m^4^C.

Each set of protein sequences was aligned using Muscle v. 5.1 [19] run under Geneious Prime 2023.0.4 (https://www.geneious.com) using default parameters. An HMM was built from each alignment using Hmmer v. 3.3.2 hmmbuild (http://hmmer.org). A list of the HMMs can be found in the Supplementary Materials, Table S1. Of the 62 HMMs, 41 were built from the MTases and 21 from the other functions.

### 2.4. Bacterial Genes and Genomes

For comparison with archaea, we also retrieved the set of RM genes encoded in all completely sequenced bacterial genomes deposited in REBASE that had associated methylome data, resulting in a total of 36,718 RM-related genes from 3369 genomes. These RM-related genes were individually classified using the same HMM library and methodology used for the archaeal genomes described above. 

### 2.5. Characterization of MTase Activity

Plasmid clones were synthesized (GenScript Biotech, Piscataway, NJ, USA) with codon-optimized genes encoding *suaIIM* and *asp7IM* in pRRS10, a lower-copy number derivative of the constitutive expression plasmid pRRS (GenBank acc. no. JN569339) with a pBR322 origin of replication. Clones were used to transform the DNA methylation-deficient *E. coli* strain ER2796, which is notably Dcm^–^. Genomic DNA from overnight cultures grown at 37 °C in LB with 100 µg/mL ampicillin was purified using the Monarch HMW DNA Extraction Kit (New England Biolabs, Ipswich, MA, USA). DNA was sheared in a Covarys ML230 (Covarys, Woburn, MA, USA) using the 175 bp AFA-TPX protocol. 

Sequencing libraries were constructed from 100 ng of sheared DNA using the NEBNext Ultra II DNA Library Prep Kit for Illumina (New England Biolabs, Ipswich, MA, USA) and partially deaminated using the RIMS-seq2 protocol [20]. Five µL of USER-treated library DNA was used for the PCR amplification step (6 cycles, with barcoded primers from the NEBNext 96 Unique Dual Index Primers) (New England Biolabs, Ipswich, MA, USA).

Libraries were sequenced on a NextSeq (Illumina, San Diego, CA, USA) using the 2 × 76 + 8 + 8 protocol. 1.4 × 10^7^ reads from the *asp7IM* clone and 1.5 × 10^7^ reads from the *suaIIM* clone were obtained. Methylation at m^5^C sites was determined by comparing the C>T deamination rates of read1 and read2 [21]. Motifs were determined by searching for over-represented sequences around these sites using pipelines based on both MoSDi [22] and DiNAMO [23], with similar results. The presence of *dcm-6*, the nonsense mutation inactivating the *dcm* gene in the ER2796 host, was verified in the sequence assembly.

## 3. Results and Discussion

### 3.1. Archaeal Genomes and RM Genes in REBASE

Genome sequences from archaea, and the RM system-related genes encoded by them, were obtained from the REBASE database [3]. To minimize our chances of making assumptions based on missing data, we restricted our analysis to those genomes that appeared to be completely sequenced, closed, and finished—a total of 519. The genomes in this set are not evenly distributed across the phylogenetic tree, with 480 (92.5%) coming from just six archaeal classes (phylum in parentheses): Thermoprotei (Crenarchaeota); Methanomada, Halobacteria, Methanomicrobia, and Thermococci (all Euryarchaeota); and Nitrososphaerota (TACK group). This uneven distribution likely reflects a combination of sampling bias, academic or industrial interest, and ease of culturing. In the 519 archaeal genomes, we identified 4135 RM-related genes, which were grouped into 1034 sequence clusters based on 30% protein sequence identity. The sizes of these clusters ranged from 167 to 1, with 88 clusters of size ≥ 10 and 494 of size = 1. A complete list of genomes and cluster members can be found in the Supplementary Materials, Table S2.

### 3.2. Functional Categorization of Gene Clusters

For functional prediction, we constructed a library of 62 HMMs, each built from an RM-related evolutionary or functional group of protein sequences, using experimentally characterized examples where available (see Section 2). Each HMM was assigned to one of 13 general functional categories based on RM system type and biochemical activity (Supplementary Materials, Table S1). The protein sequence of the centroid of each gene cluster was used as a query to search the HMM library, and the predicted function of the cluster was determined as the functional category of the top HMM hit. For Type II DNA MTase clusters that included experimentally characterized members (largely based on SMRT sequencing data), the target site of the characterized examples was taken as representative of the entire cluster.

For each high-level taxonomic group (phylum, class, and order) represented in our set of 519 archaeal genomes, we determined the mean number of genes per genome from each of these 13 functional categories (Table 1). Looking at the set of genomes in its entirety, the most common category is Type II MTases (IIM), with about 2.7 per genome. The mean number of known Type II REase genes (IIR) is more than 30-fold lower; this partially reflects the prevalence of orphan MTases, which are similar to those of Type II RM systems but lack an REase partner. However, it is worth noting that Type IIR genes are difficult to identify based on sequence similarity [24], and our HMM library captured only three specific homologous groups of these enzymes, typified by BsiHKI, DpnII, and (presumably) DUF3883. As a result, this category is expected to be significantly under-represented in our data, with most IIR genes instead captured in the “Other” category. Type I and Type IIG RM systems are the next most common types, at just under one per genome. Type III and IV systems are the least common, at less than 0.2 per genome on average. However, it is also possible that Type IV systems are under-represented for the same reason as Type II REases.

**Figure 1:**
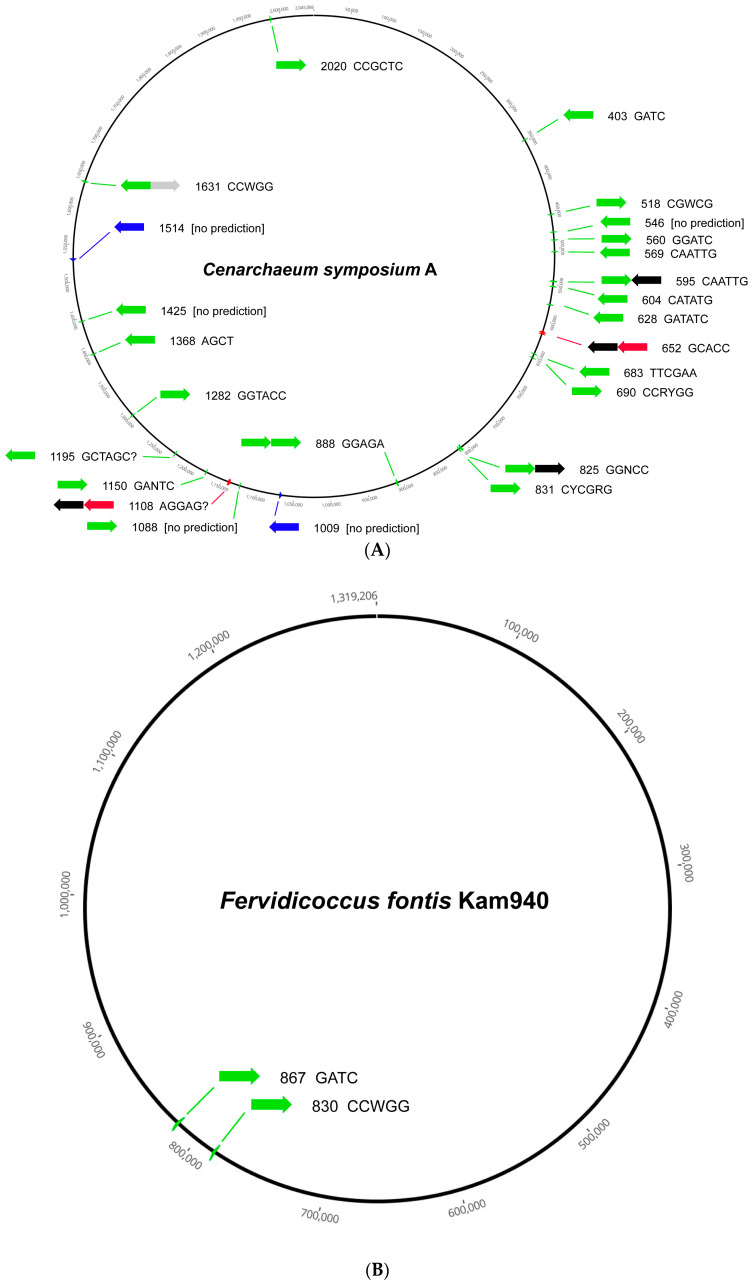
Locations of RM systems in two Crenarchaeota. For each locus, the arrows show the gene arrangement (green = Type II MTase; blue = Type IIG RM system; red = Type III MTase; black = REase; and gray = Vsr nuclease). Numerals show the ORF number from the REBASE nomenclature (where, for example, 1514 = CysAORF1514P) and the motif is the predicted recognition site based on characterized homologs. (**A**) *Cenarchaeum symbiosum* A (2.05 Mbp). (**B**) *Fervidicoccus fontis* Kam940 (1.32 Mbp).

Among the phyla, the Crenarchaeota are generally depleted in RM systems of all types, although the single representative from the order Cenarchaeales, *Cenarchaeum symbiosum* A, harbors 22 MTase genes of Type IIM, so this is not universally true. Figure 1 illustrates two extremes in RM system content in the Crenarchaeota, and archaea in general. The 25 RM system loci in *C. symbiosum* A, which are spread throughout the chromosome, include 17 orphan Type II MTases, one Type II MTase paired with a second MTase, one Type II MTase paired with a *vsr* gene, two complete Type II RM systems, two Type IIG genes, and two complete Type III RM systems (Figure 1A). All or nearly all recognize different sites based on characterized homologous examples. The genome of *Fervidicoccus fontis* Kam940 is more typical of Crenarchaeota, with only two RM loci, both Type II orphan MTases (Figure 1B).

Type I systems are particularly prevalent among the Methanomicrobia, at more than three per genome, and Type III systems are prevalent among both the Methanomicrobia and Thermoplasmata. The Halobacteria and Methanomicrobiales are relatively rich in Type IIG RM systems, at more than one per genome. Factors affecting the differences in RM system content and type between taxonomic groups may include the frequency of exposure to phage, the relative efficiency of horizontal exchange, and the microbiomes in which their members typically reside. 

### 3.3. DNA Methylation Phenotypes

Of the 519 complete archaeal genomes under consideration here, 49 have associated methylome data from SMRT sequencing (Pacific Biosciences). From these data, one can readily identify DNA motifs around m^6^A and m^4^C methyl marks; m^5^C-associated motifs can also sometimes be identified, but with less efficiency and accuracy [25,26]. Alternative methods such as bisulfite sequencing, EM-seq [27], TAPS-seq [28], and RIMS-seq [21] are better suited to identifying m^5^C motifs, but they have not yet been applied to archaeal genomes at a large scale. Table 2 shows the number of genomes in each taxonomic group that have associated methylome data derived from SMRT sequencing, as well as the mean number of genes and observed motifs of each methylation type.

It is expected that the number of MTase genes should equal or exceed the number of motifs since not every gene is active, and many m^5^C motifs are not detected via SMRT sequencing. Indeed, we observed that in general, the numbers of genes and motifs are comparable, indicating that most of the MTase genes are active. We observed two cases where the number of motifs exceeds the number of genes: m^6^A in Desulfurococcales and m^4^C in Methanosarcinales (Table 2). This can be due to erroneous prediction of protein activities (typically misclassifying m^6^A vs. m^4^C) or identification of motifs (typically misclassifying m^4^C vs. m^5^C), or it may indicate that the genome sequence is incomplete, likely missing one or more plasmids that could encode additional MTases. In the archaea as a whole, the ratio of MTase genes predicted to encode m^6^A, m^4^C, and m^5^C enzymes is approximately 4:2:1 (Table 2). Certain phyla show significantly different ratios, however. In Crenarchaeota, the most prevalent class is m^5^C due to the universal presence of a single persistent m^5^C MTase (see below) and the general depletion of RM systems in this taxon. In the TACK group, the most prevalent class is m^4^C due to the presence of several persistent m^4^C MTases in the Nitrososphaerota (see below). 

### 3.4. Comparison with Bacteria

**Figure 2:**
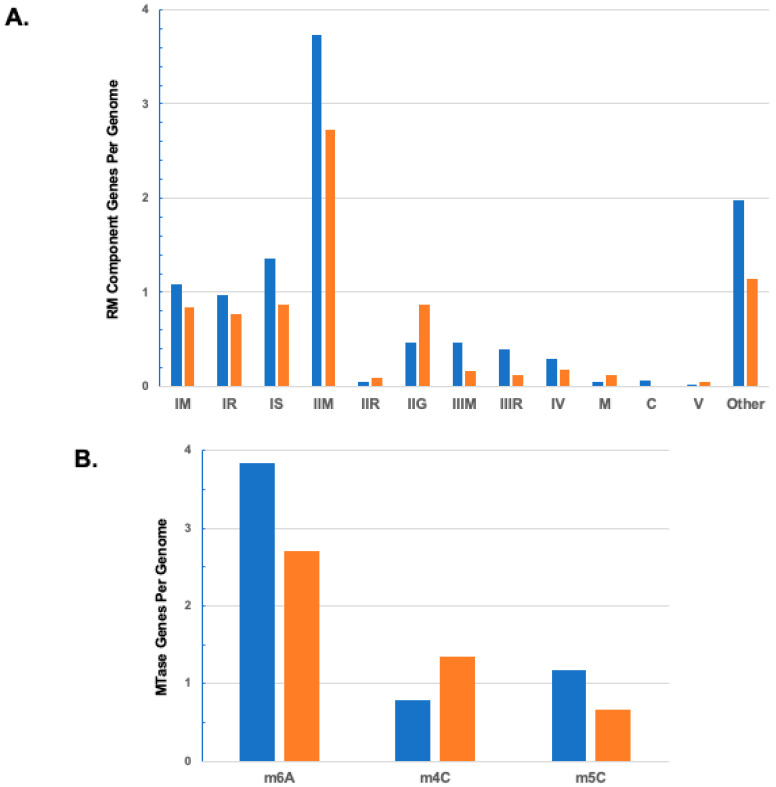
(**A**) Mean numbers of RM system genes, by function, in 3369 bacterial genomes (blue) and 519 archaeal genomes (orange). Values for archaea and definitions of the functional groups are from Table 1. (**B**) Mean numbers of MTase genes conferring each of the three methylated bases in the same sets of bacterial and archaeal genomes. Values for archaea are from Table 2.

For comparison with archaea, we retrieved a large set of completely sequenced bacterial genomes from REBASE and performed a similar analysis (see Section 2). Overall, archaea encoded fewer RM-related genes than bacteria (7.9 vs. 10.9), and this was true of every class of genes except IIR, IIG, M (BREX), and V (Figure 2A). Interestingly, the overall ratio of m^6^A, m^4^C, and m^5^C MTase genes in the bacterial genome set is approximately 5:1:1.5, with m^5^C outnumbering m^4^C (Figure 2B). The relative difference in the ratio of m^4^C and m^5^C between bacteria and archaea may reflect a greater proportion of hyperthermophiles in archaea.

### 3.5. Persistent MTases and RM Systems

Many RM systems and orphan MTases show a “patchy” distribution of homologs across a phylogenetic tree and significant differences between closely related strains, a pattern most parsimoniously explained by frequent horizontal gene transfer (HGT) and gene loss [13]. The resulting diversity of defense systems can be advantageous in protecting a population from infection by phage and other deleterious genetic elements. However, the ability of DNA methylation to affect gene transcription and other DNA–protein interactions can result in orphan DNA MTases (and sometimes full RM systems) acquiring functional roles outside of cellular defense. When this happens, the selective pressure on the genes encoding them can favor conservation and vertical transmission; such genes are sometimes termed “persistent” because they are less likely to be lost over time than most RM systems [11]. Classical examples of these include *dam* in the Gammaproteobacteria and *ccrM* in the Alphaproteobacteria. Large-scale comparative genomic studies have identified additional examples in bacteria and in the archaeal phylum Halobacteria [11,13,14,29].

We define a persistent MTase or RM system as one that is present in at least 75% of members of a given taxonomic group represented by at least five genomes in our set. We mapped the 88 clusters with ≥10 members to the taxonomic tree of the 519 archaeal genomes to identify such cases. For those clusters that met our definition, or nearly so, we combined them with closely related clusters, built phylogenetic trees on the combined sets, and reassorted the members based on monophyletic groups where necessary. We refer to these manually adjusted clusters as homologous groups (HGs). Table 3 shows each taxonomic group encoding at least one HG that met the criteria for persistence, and Supplementary Materials Table S3 shows the original cluster number to which each HG member belongs.

We identified 1 persistent group at the phylum level, 3 at the class level, 7 at the order level, and 18 between the levels of family and species. Of these 29 persistent systems, 20 are Type II orphan MTases (all with 4–5 base recognition sites, and all but one palindromic), 1 is a complete Type II RM system, 2 are BREX-like MTases, 5 are Type I systems (comprising 2 or 3 genes), and 1 is a Type IV REase (Table 3). Four persistent systems (HG2, HG3, HG1, and HG11) are shared between multiple taxonomic groups, which may be due either to independent acquisition or to gene loss in sister taxa.

The largest group, HG1, is found throughout the Halobacteria (163/181), except for *Halorubrum* (1/10) and *Haloquadraticum* (0/2); its members are orphan m^4^C MTases that modify CTAG (with the underline here and elsewhere indicating the methylated base), and it corresponds to cHG U observed previously by Fullmer and coworkers [13]. Although the general function of this epigenetic signal remains unknown, the CTAG sequence is generally under-represented in Halobacterial genomes [13] but locally clustered upstream of orc6/cdc1 gene orthologs [14], which encode the origin of replication binding complex in most archaea, a role analogous to that of DnaA in bacteria. This suggests a role for HG1 in chromosome replication or the regulation thereof in Halobacteria, but its precise function remains to be elucidated.

The second largest group, HG2, is found almost universally throughout the Crenarchaeota phylum (99/100) as well as in most Methanococci (where in *Methanocaldococcus* it is present in two copies) and *Pyrococcus*; its members are orphan m^5^C MTases. Prior to this work, two examples from this clade had predicted recognition sites, although neither had been tested directly: M.SuaII had been predicted to modify RGATCY based on SMRT sequencing of *Sulfolobus acidocaldarius* DSM639 [14] and M.Asp7I was predicted to modify GGCAC in *Acidilobus* species 7A. To address the conflicting predictions, we cloned and expressed both genes in a methyl-deficient strain of *E. coli* and, using RIMS-seq [21], found both to modify the heterologous host chromosome in vivo at CCWGG sites, the same site modified in wild-type *E. coli* strains by the product of *dcm*. In other words, both predictions were incorrect. The presence of a persistent m^5^C MTase in hyperthermophiles is intriguing since the rate of deamination of m^5^C is expected to be high at elevated temperatures, leading to a mutator phenotype [30]. The answer to this conundrum may be that HG2 is silenced under most conditions: although M.SuaII is active as a constitutively expressed clone, negligible levels of m^5^C methylation were observed in its native host, *S. acidocaldarius*, under the conditions of one published experiment [31]. This suggests that HG2 may be under tight regulatory control, in contrast to Dcm, which provides nearly complete methylation of CCWGG sites in *E. coli*.

The third largest group, HG3 (which corresponds to cHG W described previously [13]), encodes a Dam-like orphan m^6^A MTase that, based on characterized examples, modifies GATC sites. This MTase appears to have been independently established in several taxa: genus *Methanobacterium* (10/12), species *Methanococcus maripaludis* (9/9), family Halorubraceae (15/20, often accompanied by a second, plasmid-encoded copy), order Methanomicrobiales (15/19), and class Nitrososphaerota (27/29). HG3 members also sporadically appear in other strains, sometimes as an orphan and sometimes with an associated REase gene.

Group HG4, nearly ubiquitous in Natrialbales (41/42), encodes an orphan m^6^A MTase and is the only example of a Type II MTase group found here that modifies a nonpalindromic sequence, CATTC. All of the remaining persistent Type II MTases modify m^4^C: HG10 and HG18 (GTAC); HG11, HG16, and HG19 (AGCT); HG12 (CGCG); HG15 (GGCC); HG20 (CTNAG); and HG21 (unknown recognition site). All are orphans except for HG15, which is always accompanied by a companion REase, an arrangement atypical of persistent systems [11]. Two taxa, the Methanomicrobiales and the Nitrososphaerota, are particularly rich in these persistent m^4^C orphan MTases. Interestingly, in both taxa, GATC (conferred by HG3) and AGCT (conferred by HG11, HG16, or HG19) are present throughout the group or nearly so, with one or more additional persistent m^4^C groups present in the subclades. This may indicate a common epigenetic function for GATC and AGCT methylation in these distantly related taxa.

Several Type I RM systems met the criteria for persistence. However, given that the target sites of these systems are dictated by the specificity subunit, which tends to be the least conserved of the three Type I components, it is not clear that members of all of these systems recognize and methylate the same sequence. It may be that these systems are not vertically inherited, but rather are frequently horizontally exchanged between strains of the same species or taxon. HG6, for example, is also found frequently in Thermococcus and Methanothermobacter, and HG14 in other Methanomicrobia. Interestingly, four of the five Type I RM systems that meet the criteria for persistence are found in Methanosarcina, and they are the primary reason that this species has the highest density of Type I RM systems in the archaea generally, at more than four per genome (Table 1). 

HG5 resembles PglX, the MTase associated with BREX systems, and is persistent in the genus Haloterrigena but sporadically found throughout the rest of the Halobacteria. HG13, which weakly resembles Eco57I-like Type IIG systems, is persistent in *Methanosarcina mazei* (where it is largely coincident with the four Type I systems) but sporadic throughout other Methanosarcinales. The lone persistent Type IV system, HG9, strongly resembles (39% identity) Mrr from *E. coli* K-12 and is persistent in the genus *Methanosarcina* (22/29).

The determination of persistence is highly dependent on the availability of completely sequenced genomes. Many taxa in our set are not represented by a sufficient number of genomes to be able to determine persistence based on our criteria. In general, higher-order taxa are represented by more examples than lower-order taxa. However, even among higher-order taxa, two of six phyla and 9 of 16 classes are represented in our set by fewer than five examples, too few to make a persistence determination. Also, in general, lower-order taxa tend to be less diverse groups and therefore would be expected to have more persistent systems than higher-order taxa. However, more specific taxa are also less likely to have enough examples to make the assessment. For example, only seven named archaeal species have more than five examples in our set, but three of these seven have at least one persistent system by our criteria (Table 3). The sequencing to closure of additional archaeal genomes from a broad diversity of taxa will no doubt reveal many additional examples.

## Figures and Tables

**Table 1:** Mean number of RM-related genes per taxonomic group.

Taxonomic Group ^a^	No. Genomes	IM	IR	IS	IIM	IIR	IIG	IIIM	IIIR	IV	M	C	V	Other
**Asgard group** (Lokiarchaeota)	**1**	0	0	0	11	0	4	2	0	0	0	0	0	2
**Thermoplasmatota**	**18**	0.611	0.556	0.444	3.389	0.056	0.389	0.778	0.667	0.556	0	0	0.056	1.667
Aciduliprofundum	2	0.5	0.5	0.5	1.5	0	1	0.5	0.5	0	0	0	0	1
Thermoplasmata	16	0.625	0.562	0.438	3.625	0.062	0.312	0.812	0.688	0.625	0	0	0.062	1.75
*Methanomethylophilaceae*	1	1	1	0	0	0	0	0	0	1	0	0	0	2
*Methanomassiliicoccales*	5	0.6	0.6	0.2	2	0.2	0.2	0.2	0.2	1	0	0	0	1.4
*Thermoplasmatales*	9	0.667	0.556	0.667	5	0	0.444	1.333	1.111	0.333	0	0	0.111	1.444
*Unclassified*	1	0	0	0	3	0	0	0	0	1	0	0	0	6
**Crenarchaeota** (Thermoprotei)	**100**	0.04	0.04	0.04	1.97	0.03	0.44	0.04	0.03	0	0	0	0.05	1.18
*Acidilobales*	3	0	0	0	1.333	0	0	0	0	0	0	0	0	0.667
*Cenarchaeales*	1	0	0	0	22	0	2	2	2	0	0	0	0	3
*Desulfurococcales*	17	0.059	0.059	0.059	2.294	0	0.647	0	0	0	0	0	0.118	0.824
*Fervidicoccales*	1	0	0	0	2	0	0	0	0	0	0	0	0	0
*Sulfolobales*	59	0.051	0.051	0.051	1.542	0	0.39	0.034	0.017	0	0	0	0	1.271
*Thermofilales*	5	0	0	0	2.4	0.4	0.4	0	0	0	0	0	0.2	0.8
*Thermoproteales*	14	0	0	0	1.929	0.071	0.429	0	0	0	0	0	0.143	1.429
**DPANN group**	**6**	0.333	0.167	0.5	1	0.167	0.167	0.5	0.333	0	0	0	0	0.167
Micrarchaeota	2	0	0	0.5	1.5	0.5	0	1.5	1	0	0	0	0	0
Nanohaloarchaeota (*Nanohalobia*)	1	1	1	1	1	0	1	0	0	0	0	0	0	1
Nanoarchaeota	3	0.333	0	0.333	0.667	0	0	0	0	0	0	0	0	0
**Environmental sample**	**1**	1	1	1	9	0	1	1	1	0	0	0	0	0
**Euryarchaeota**	**362**	1.124	1.033	1.157	2.798	0.113	1.064	0.152	0.130	0.227	0.180	0	0.055	1.174
Archaeoglobi (*Archaeoglobales*)	8	1	0.75	0.875	0.875	0	0.25	0.25	0.25	0	0	0	0	1.375
Methanoliparia	1	2	2	2	5	0	2	0	0	0	0	0	0	3
Methanomada	61	1	0.951	1.344	2.082	0.262	0.77	0.41	0.328	0.279	0.148	0	0.033	1.656
*Methanobacteria*	36	0.944	0.889	1.472	1.444	0.333	0.611	0.361	0.278	0.472	0.222	0	0.056	1.222
*Methanococci*	24	1.125	1.083	1.208	3.083	0.167	0.958	0.5	0.417	0	0.042	0	0	2.375
*Methanopyri*	1	0	0	0	1	0	2	0	0	0	0	0	0	0
Methanonatronarchaeia	1	0	0	0	2	0	0	0	0	0	0	0	1	0
Halobacteria	181	0.431	0.409	0.420	3.602	0.11	1.354	0.033	0.028	0.066	0.149	0	0.055	0.890
*Halobacteriales*	94	0.457	0.426	0.415	3.320	0.128	1.362	0.011	0.011	0.053	0.138	0	0.064	0.904
*Haloferacales*	45	0.444	0.422	0.489	3.533	0.089	1.4	0.067	0.044	0.133	0.089	0	0.067	0.844
*Natrialbales*	42	0.357	0.357	0.357	4.31	0.095	1.286	0.048	0.048	0.024	0.238	0	0.024	0.905
Methanomicrobia	65	3.585	3.215	3.462	2.462	0.015	0.831	0.323	0.292	0.815	0.446	0	0.077	1.138
*Methanocellales*	3	1	1	1	6	0	0.333	0.667	0.333	0	0	0	0	1.333
*Methanomicrobiales*	19	2.105	1.684	1.789	4.579	0.053	1.105	0.368	0.316	0.737	0.421	0	0.053	1.789
*Methanosarcinales*	42	4.524	4.143	4.476	1.143	0	0.738	0.286	0.286	0.905	0.5	0	0.095	0.833
*Unclassified*	1	0	0	0	7	0	1	0	0	1	0	0	0	1
Nanohaloarchaeota	1	0	0	0	1	0	0	0	0	0	0	0	0	0
Thermococci (*Thermococcales*)	44	0.568	0.568	0.614	1.341	0.091	0.795	0.023	0.023	0	0	0	0.045	1.705
**TACK group**	**31**	0.419	0.419	0.581	3.71	0	0.29	0.129	0	0	0	0	0.097	0.516
Geothermarchaeota	1	0	0	0	0	0	1	1	0	0	0	0	0	1
Korarchaeota	1	0	0	0	1	0	1	0	0	0	0	0	0	0
Nitrososphaerota	29	0.448	0.448	0.621	3.931	0	0.241	0.103	0	0	0	0	0.103	0.517
*Nitrosopumilales*	14	0.286	0.286	0.571	2.786	0	0	0	0	0	0	0	0	0.714
*Nitrososphaeria*	10	0.6	0.6	0.6	5.8	0	0.7	0.2	0	0	0	0	0.3	0.1
*Nitrososphaerota inc. sed.*	5	0.6	0.6	0.8	3.4	0	0	0.2	0	0	0	0	0	0.8
**TOTAL**	**519**	0.844	0.776	0.873	2.721	0.089	0.869	0.160	0.125	0.177	0.125	0	0.056	1.141

^a^ For the purposes of this work, the taxa in bold will be considered phyla, those in Roman type classes, and those in italics orders. If every member of a particular taxon represented here belongs to the same lower-order taxon, that lower-order taxon is shown in parentheses next to the higher-order taxon.

**Table 2:** Mean numbers of MTase genes and motifs based on methylated base and position.

	All Complete Genomes				Genomes with Methylation Data						
Taxonomic Group ^a^	Genomes	Genes m^6^A	Genes m^4^C	Genes m^5^C	Genomes	Genes m^6^A	Genes m^4^C	Genes m^5^C	Motifs m^6^A	Motifs m^4^C	Motifs m^5^C
**Asgard group** (Lokiarchaeota)	**1**	**10**	**7**	**0**							
**Thermoplasmatota**	**18**	**2.556**	**2.167**	**0.444**	**2**	**6**	**4.5**	**0**	**5**	**2.5**	**0**
Aciduliprofundum	2	2	1.5	0							
Thermoplasmata	16	2.625	2.25	0.5							
*Methanomethylophilaceae*	1	1	0	0							
*Methanomassiliicoccales*	5	1.4	1	0.6							
*Thermoplasmatales*	9	3.778	3.444	0.222	2	6	4.5	0	5	2.5	0
*Unclassified*	1	0	0	3							
**Crenarchaeota** (Thermoprotei)	**100**	**0.83**	**0.56**	**1.1**	**3**	**0.667**	**0.667**	**1**	**1**	**0.667**	**0.667**
*Acidilobales*	3	0.333	0	1	1	0	0	1	0	0	1
*Cenarchaeales*	1	13	12	1							
*Desulfurococcales*	17	1	0.882	1.118	1	2	1	1	3	1	0
*Fervidicoccales*	1	0	1	1							
*Sulfolobales*	59	0.576	0.441	1	1	0	1	1	0	1	1
*Thermofilales*	5	1	0.4	1.4							
*Thermoproteales*	14	0.929	0	1.429							
**DPANN group**	**6**	**1.333**	**0.5**	**0.167**	**1**	**1**	**0**	**0**	**1**	**0**	**0**
Micrarchaeota	2	2	1	0							
Nanohaloarchaeota (*Nanohalobia*)	1	2	0	1							
Nanoarchaeota	3	0.667	0.333	0	1	1	0	0	1	0	0
**Environmental sample**	**1**	6	6	0							
**Euryarchaeota**	**362**	**3.282**	**1.442**	**0.594**	**39**	**3.538**	**1.615**	**0.641**	**2.538**	**1.308**	**0.103**
Archaeoglobi (*Archaeoglobales*)	8	1.625	0.375	0.375							
Methanoliparia	1	4	5	0							
Methanomada	61	3.033	0.574	0.803	5	4	1.2	1.2	3.4	1	0.2
*Methanobacteria*	36	2.806	0.25	0.528	3	3	0.333	1	2.333	0.333	0
*Methanococci*	24	3.417	1.083	1.208	2	5.5	2.5	1.5	5	2	0.5
*Methanopyri*	1	2	0	1							
Methanonatronarchaeia	1	0	1	1							
Halobacteria	181	2.856	2.055	0.657	26	3.462	1.769	0.615	2.461	1.269	0.077
*Halobacteriales*	94	2.777	1.872	0.638	10	3.1	1.2	0.4	1.6	1.1	0.1
*Haloferacales*	45	3.2	1.556	0.778	10	3.5	1.5	0.9	2.9	1.1	0.1
*Natrialbales*	42	2.667	3	0.571	6	4	3.167	0.5	3.167	1.833	0
Methanomicrobia	65	6.031	1.277	0.338	4	5	2.5	0.25	2.5	3	0.25
*Methanocellales*	3	4.667	2.667	0.667							
*Methanomicrobiales*	19	5.316	2.895	0.368	3	6	3	0.333	3	3.333	0.333
*Methanosarcinales*	42	6.548	0.381	0.262	1	2	1	0	1	2	0
*Unclassified*	1	2	4	2							
Nanohaloarchaeota	1	0	1	0							
Thermococci (*Thermococcales*)	44	1.75	0.5	0.477	4	2	0.25	0.5	2	0.25	0
**TACK group**	**31**	**2.065**	**2.129**	**0.355**	**4**	**1.5**	**1.5**	**0.5**	**1.5**	**1.25**	**0.25**
Geothermarchaeota	1	2	0	0							
Korarchaeota	1	1	1	0							
Nitrososphaerota	29	2.103	2.241	0.379	4	1.5	1.5	0.5	1.5	1.25	0.25
*Nitrosopumilales*	14	1.643	1.071	0.357	3	1.667	1	0.667	1.667	1	0.333
*Nitrososphaeria*	10	2.8	4.1	0.4	1	1	3	0	1	2	0
*Nitrososphaerota inc. sed.*	5	2	1.8	0.4							
**TOTAL**	**519**	2.707	1.347	0.665	49	3.245	1.633	0.612	2.429	1.286	0.143

^a^ For the purposes of this work, the taxa in bold will be considered phyla, those in Roman type classes, and those in italics orders. If every member of a particular taxon represented here belongs to the same lower-order taxon, that lower-order taxon is shown in parentheses next to the higher-order taxon.

**Table 3:** Persistent RM systems in each taxonomic group.

Taxonomic Group ^a^	Total Genomes	Cluster (Members)	Class	Motif ^b^
**Thermoplasmatota**	**18**	**None**		
**Crenarchaeota** (Thermoprotei)	**100**	**HG2 (99)**	**IIM**	**CCWGG (m^5^C)**
*Sulfolobales*	59	None		
*Sulfolobus acidocaldarius*	9	HG15 M/R (8)	IIM/R	GGCC (m^4^C)
**DPANN group**	**6**	**None**		
**Euryarchaeota**	**363**	**None**		
Archaeoglobi (*Archaeoglobales*)	8	HG6 M/R (6)	IM/R	n/d
Methanomada	61	None		
*Methanobacteria*	36	None		
*Methanobacterium*	12	HG3 (10)	IIM	GATC (m^6^A)
*Methanococci*	24	HG2 (19)	IIM	CCWGG (m^5^C)
*Methanococcus maripaludis*	9	HG3 (9)	IIM	GATC (m^6^A)
Halobacteria	182	HG1 (155)	IIM	CTAG (m^4^C)
*Haloferacales*	45	None		
*Halorubraceae*	20	HG3 (15)	IIM	GATC (m^6^A)
*Natrialbales*	42	HG4 (41)	IIM	CATTC (m^6^A)
*Haloterrigena*	9	HG5 (7)	BREX	CTGGAG (m^6^A)
Methanomicrobia	65	None		
*Methanomicrobiales*	19	HG3 (15)	IIM	GATC (m^6^A)
*Methanoculleus*	6	HG16 (6)	IIM	AGCT (m^4^C)
*M-regula/M-spirilla group* ^c^	6	HG16 (6)	IIM	AGCT (m^4^C)
*M-regula/M-spirilla group* ^c^	6	HG18 (6)	IIM	GTAC (m^4^C)
*M-regula/M-spirilla group* ^c^	6	HG20 (6)	IIM	CTNAG (m^4^C)
*Methanosarcinales*	42	HG8 M/R/S (28)	IM/R/S	n/d
*Methanosarcina*	29	HG9 (22)	IV	n/d
*Methanosarcina mazei*	9	HG17 M/R (7)	IM/R	n/d
*Methanosarcina mazei*	9	HG13 (7)	BREX	n/d
*Methanosarcina mazei*	9	HG14 M/R (9)	IM/R	n/d
*Methanosarcina mazei*	9	HG7 M/R/S (9)	IM/R/S	n/d
Thermococci (*Thermococcales*)	44	None		
*Pyrococcus*	9	HG2 (9)	IIM	CCWGG (m^5^C)
**TACK group**	**31**	**None**		
Nitrososphaerota	29	HG3 (27)	IIM	GATC (m^6^A)
*Nitrosopumilales*	14	HG11 (14)	IIM	AGCT (m^4^C)
*Nitrososphaeria*	10			
*Nitrososphaerales*	8	HG10 (8)	IIM	GTAC (m^4^C)
*Nitrososphaerales*	8	HG19 (8)	IIM	AGCT (m^4^C)
*Nitrososphaera*	5	HG12 (5)	IIM	CGCG (m^4^C)
*Nitrososphaera*	5	HG21 (5)	IIM	Unknown
*Nitrososphaerota inc. sed.*	5	HG11 (4)	IIM	AGCT (m^4^C)

^a^ For the purposes of this work, the taxa in bold will be considered phyla, those in Roman type classes, and those in italics orders. If every member of a particular taxon represented here belongs to the same lower-order taxon, that lower-order taxon is shown in parentheses next to the higher-order taxon. ^b^ Methylated base on the top strand is underlined. ^c^ Methanospirillaceae and Methanoregulaceae consistently form a subclade under Methanomicrobiales and are treated as a single group for the purpose of this table.

## Data Availability

Publicly available datasets were analyzed in this study. These data can be found in REBASE (rebase.neb.com) [3].

## Supplementary Materials

The following supporting information can be downloaded at: https://www.mdpi.com/article/10.3390/microorganisms11102424/s1. Table S1: Profiles and functions used for gene classification. Functions refer to the biochemical function(s) of the proteins in the set: M = MTase; R = REase; C = control protein (transcriptional regulator); S = target specificity; V = Vsr repair endonuclease. Categories refer to the 13 general functional categories, a combination of biochemical activity and RM system type, used for functional classification. The methylation type applies only to the MTases. Table S2: Number of protein cluster members in each genome. In the header are shown the cluster number, number of members, and top HMM hit to the cluster centroid. Columns are in descending order by cluster size. Entries show the number of cluster members (*n*) in each genome, colored pink when *n* = 1 and yellow when *n* > 1. Those genomes with associated methylome data from SMRT sequencing are shaded in green. Orgnum = REBASE organism number; taxonomy = taxonomy string as determined by NCBI. Table S3: Number of homologous group (HG) members in each genome, showing only those homologous groups that are persistent in at least one taxon. In the header are shown the HG number, number of members, and top HMM hit to the cluster centroid. Columns are in descending order by HG size, except for Type I RM systems, for which the persistent MTase member is shown adjacent to its companion REase and specificity subunits, which may or may not also be persistent. Members of the same RM system are shaded in color in the top row. Entries show the original cluster number of each HG member(s), colored in orange for those taxa where it meets the criteria for persistence. Those genomes with associated methylome data from SMRT sequencing are shaded in green. Orgnum = REBASE organism number; taxonomy = taxonomy string as determined by NCBI.
