# Supplementary material for: A Survey of Archaeal Restriction–Modification Systems
Source: Microorganisms. 2023 Sep 28;11(10):2424. doi: 10.3390/microorganisms11102424 (PMC10609329; doi:10.3390/microorganisms11102424)
Supplement: Supplementary file 1 [file microorganisms-11-02424-s001.zip › Table_S1.pdf]

Supplementary Table S1. Profiles and functions used for gene classification.

Profile Name	Function(s)	Category	Methylation	Description	No. Sequences
a1a	M	IIM	m^6A	MTases, group A1 (m^6A)	23
a1c	M	IIM	m^4C	MTases, group A1 (m^4C)	8
a2	M	IIM	m^6A	MTases, group A2	16
b-cog1743	R, M (?)	IIG	m^6A	Annotated as COG1743 (N-MTase with Zn ribbon), probable Type IIG, distantly related to B3 and B4; no characterized examples	8
b1a	M	IIIM	m^6A, m^4C	Type III Mod genes, mostly m^6A but a few m^4C	38
b1k	M	IIM	m^6A	Type III-like Type II MTases (e.g., KpnI)	3
b2a	M	IIM	m^6A	MTases, group B2 (m^6A)	6
b2c	M	IIM	m^4C	MTases, group B2 (m^4C); those with bipartite recognition sites have long insertions	25
m1bbs	M	IIM	m^4C	MTases, group B2, probably permuted from b2C ancestor to alpha or gamma (e.g., M1.BbsI)	4
b3	M	IIM	m^4C, m^6A	MTases, group B3, probably permuted from b2C ancestor to alpha; all m^4C except M.BspHI	22
b4	M	IIM	m^6A	MTases, group B4, probably permuted from b2A ancestor, with Zn ribbon; uncharacterized except M.NdeI	4
b4btgz	R, M	IIG	m^6A	BtgZI-like Type IIG, accompanied by 2 MTases; Mrr-like REase domain at C-terminus	8
c1bot	M	IIM	m^5C	MTases, m^5C	38
c1top	M	IIM	m^5C	MTases, m^5C	30
ecori-like	M	IIM	m^6A	MTases, M.EcoRI-like	6
g1a	M	IM	m^6A	Type Ia HsdM	14
g1b	M	IM	m^6A	Type Ib HsdM	7
g1c	M	IM	m^6A	Type Ic HsdM	12
g1d	M	IM	m^6A	Type Id HsdM	17
g1e	M	IM	m^6A	Type Ie HsdM	8
g2-bcg	R, M	IIG	m^6A	BcgI-like Type IIG, accompanied by separate S gene; cleaves on both sides at 13/11	7
g2-haeiv	R, M	IIG	m^6A	HaeIV-like Type IIG, single RMS protein; cleaves on both sides at 13/7 and 14/9	8
g2	M	IIM	m^6A	MTases, group G2; all with split recognition sites	15
g3-drdv	R, M	IIG	m^6A	DrdV-like Type IIG; RM with SF2 helicase domain, cleaves randomly; S-like C-terminal domain	7
g3-tspgw	R, M	IIG	m^6A	TspGW-like Type IIG; similar to DrdV but no helicase, cleaves 11/9; S-like C-terminal domain	5

g4-mme	R, M	IIG	m^6A	MmeI-like Type IIG; cleaves 20/18	15
g5	M	IIM	m^6A	MTases, group G5; all contiguous recognition sites, prototype M.Hpy99XII	7
g6	M	IIM	m^6A	MTases, group G5; all contiguous recognition sites, prototype M.Hpy188III	5
g7	M	IIM	m^6A	MTases, group G7; all contiguous recognition sites	25
g7-bsmf	R, M	IIG	m^6A	BsmFI-like Type IIG, accompanied by 2 MTases; REase domain at N-terminus; cleaves 10/14	5
g7-bspcn	R, M	IIG	m^6A	BspCNI-like Type IIG, accompanied by 1 MTase; cleaves 9/7	6
g7-eco57	R, M	IIG	m^6A	Eco57I-like Type IIG, accompanied by 1 MTase; cleaves 16/14	7
g7-tth111	R, M	IIG	m^6A	Tth111II-like Type IIG, accompanied by 1 MTase (if non-palindromic recognition site); cleaves 11/9	9
g8-pglx	M	M	m^6A	BREX-like MTases	10
lmoa118-like	M	IIM	m^6A, m^4C	MTases, mini-gamma configuration, includes M.LmoA118I (m^6A) and M.NgoMXV (m^4C)	2
mun-like	M	IIM	m^6A	MTases, M.MunI-like; derived from m^6A RNA MTases	5
nru-like	M	IIM	m^6A, m^4C	MTases, gamma with DPPY motif IV; includes M.NruI (m^6A) and M.Sbo13I (m^4C)	4
snab-like	M	IIM	m^4C	MTases, M.SnaBI-like	5
t1dam-like	M	IIM	m^6A	MTases, M.T1Dam-like (phage-encoded)	4
m2maeiv	M	IIM	m^6A	MTases, M2.MaeIV-like, accompany Type IIG systems	4
bth571i	R, M	IIG	m^6A	Bth571I-like Type IIG, sometimes accompanied by a complementing MTase fragment	10
bsihki	R	IIR	n/a	REases, BsiHKAI-like	6
cgenes	C	C	n/a	C genes	13
dpnii	R	IIR	n/a	REases, DpnII-like	7
iiir-gp1	R	IIIR	n/a	Type III Res, EcoPI-like	17
iiir-gp2	R	IIIR	n/a	Type III Res, associated with m^4C Mod	7
iiir-gp3	R	IIIR	n/a	Type III Res, PstII-like	4
iiir-gp4	R	IIIR	n/a	Type III Res, Xor86I-like	9
iv-mrr	R	IV	n/a	Type IV REases, EcoKMrr-like	13
iv-ri-like	R	IV	n/a	Type IV REases, ScoA3I-like	4
pdexk-duf3883	R (?)	IIR	n/a	Presumed REases (PDDEXK nucleases, DUF3883); no characterized examples	7
r1a	R	IR	n/a	Type Ia HsdR	13
r1b	R	IR	n/a	Type Ib HsdR	7
r1c	R	IR	n/a	Type Ic HsdR	12
r1d	R	IR	n/a	Type Id HsdR	15
r1e	R	IR	n/a	Type Ie HsdR	7
s1a	S	IS	n/a	Type Ia HsdS	14
s1b	S	IS	n/a	Type Ib HsdS	6
s1c	S	IS	n/a	Type Ic HsdS	7

s1d	S	IS	n/a	Type Id HsdS	16
s1e	S	IS	n/a	Type Ie HsdS	12
vgenes	V	V	n/a	Vsr nucleases (mismatch repair nicking enzymes)	6
